# Vaccine-Preventable Hospitalisations from Seasonal Respiratory Diseases: What Is Their True Value?

**DOI:** 10.3390/vaccines11050945

**Published:** 2023-05-04

**Authors:** Margherita Neri, Simon Brassel, Hannah Schirrmacher, Diana Mendes, Andrew Vyse, Lotte Steuten, Elizabeth Hamson

**Affiliations:** 1 Office of Health Economics, London SE1 2HB, UK; 2 Triangulate Health, Doncaster DN11 9QU, UK; 3 Pfizer Ltd., Tadworth KT20 7NT, UK

**Keywords:** opportunity cost, hospitalisation costs, respiratory infections, vaccine-preventable hospitalisations, vaccines value

## Abstract

Hospitals in England experience extremely high levels of bed occupancy in the winter. In these circumstances, vaccine-preventable hospitalisations due to seasonal respiratory infections have a high cost because of the missed opportunity to treat other patients on the waiting list. This paper estimates the number of hospitalisations that current vaccines against influenza, pneumococcal disease (PD), COVID-19, and a hypothetical Respiratory Syncytial Virus (RSV) vaccine, could prevent in the winter among older adults in England. Their costs were quantified using a conventional reference costing method and a novel opportunity costing approach considering the net monetary benefit (NMB) obtained from alternative uses of the hospital beds freed-up by vaccines. The influenza, PD and RSV vaccines could collectively prevent 72,813 bed days and save over £45 million in hospitalisation costs. The COVID-19 vaccine could prevent over 2 million bed days and save £1.3 billion. However, the value of hospital beds freed up by vaccination is likely to be 1.1–2 times larger (£48–93 million for flu, PD and RSV; £1.4–2.8 billion for COVID-19) when quantified in opportunity cost terms. Considering opportunity costs is key to ensuring maximum value is obtained from preventative budgets, as reference costing may significantly underestimate the true value of vaccines.

## 1. Introduction

In England, the National Health System (NHS) has a long history of excess demand in hospital settings. This is exemplified by the backlog of elective care procedures predating the COVID-19 pandemic [1]. However, the COVID-19 pandemic significantly exacerbated this situation. In October 2022, the backlog of patients waiting to receive treatment achieved a record high of 7.2 million [2]. On average, patients in the backlog are also waiting longer: in 2021/22, around 40% of those waiting to start non-urgent care waited longer than the recommended threshold of 18 weeks [3].

The winter season poses additional pressure on the already strained NHS resources, particularly in the hospital settings. In December 2022, the NHS saw a record figure of 2.3 million accident and emergency (A&E) attendances [4], driven by surges in hospitalisations due to respiratory diseases. In the first week of January 2023, England’s average hospital bed occupancy rose to 94.6% (3.2 percentage points higher than the same week in the previous year) (ibid). 

Immunisation programmes targeting communicable diseases with a high risk of hospitalisation can play a key role in mitigating this pressure on the NHS. By preventing illness and severe health outcomes leading to hospitalisations, vaccines avert the use of healthcare resources to treat individuals who are directly and indirectly protected by them. This frees up hospital capacity, which the health system can use to deliver health care to other patients. In fact, under the current circumstances of severe excess demand for hospital beds, vaccine-preventable hospitalisations always represent a missed opportunity to treat other patients on the waiting list. In other words, each vaccine-preventable hospitalisation bears an opportunity cost. 

The value generated by vaccines is inherently associated with the opportunity cost of vaccine-preventable hospitalisations. There are many ways to value opportunity costs in economics [5], such as the cost incurred for the chosen treatment or different measures of what is lost by forgoing alternative treatment opportunities. For pragmatic reasons, the most common approach proxies the opportunity costs of healthcare resources through the direct costs of the treatment they allow to deliver [5]. In the case of vaccines, this approach would consider the direct cost of admitting and treating patients in hospitals for vaccine-preventable conditions. This so-called reference costing approach equals the true opportunity costs under very re-strictive conditions (e.g., perfect competition) and might be a good enough proxy in times of no excess demand. However, its appropriateness may be compromised when there is severe excess demand for hospital beds to treat other patients from the waiting list [6,7].

To ensure that healthcare budgets for prevention are allocated to programmes that can best support the NHS recovery and enhance resilience for future winters, it is important that local and national decision-makers are well-informed about the full value generated by vaccines. However, due to the conventional use of reference costing methods to value scarce healthcare resources, there is a risk that the true value of vaccines is misrepresented.

This paper provides evidence of the volume of hospitalisations due to respiratory disease that current vaccines against influenza (flu), pneumococcal disease (PD), COVID-19, and a hypothetical Respiratory Syncytial Virus (RSV) vaccine, could prevent in the winter among older adults in England. These diseases are all leading causes of older adult respiratory hospitalisation in England in the winter months when the NHS experiences extreme pressure due to severe excess demand [8,9]. We estimated and compared their value using reference costing and an alternative opportunity costing approach to explore to what extent their value is appreciated by each. Additionally, we explored replacing the currently used PD vaccine with a new 20-valent pneumococcal conjugate vaccine (PCV20) that is now licensed for use in adults in the UK.

## 2. Methods

The empirical approach of this paper is based on prior work by Sandmann et al. [6] and Brassel et al. [10]. In their research, hospital beds are assumed to be the key resource facilitating access to hospital treatment and subject to excess demand. 

We developed a model to estimate the number of hospitalisations that current vaccination programmes against flu, PD, COVID-19, and a hypothetical RSV vaccine, could prevent in the winter among older adults (Section 2.1). We then estimated their value according to the conventional reference costing approach and an alternative opportunity costing approach (Section 2.2.).

### 2.1. Vaccine-Preventable Hospitalisations 

To model the number of vaccine-preventable hospitalisations in the winter months (October to March), we considered historical hospital admissions data pre-COVID-19 separately because non-pharmaceutical interventions (i.e., winter lockdowns) also reduced the occurrence of other respiratory diseases. Hence, for flu, PD and RSV, we considered pre-COVID-19 hospitalisation patterns from 2018/19. For COVID-19, we considered 2021/22 hospitalisation patterns.

Where available, we estimated the impact of vaccination programmes recommended by JCVI to NHS patients for the relevant years. For PD, we modelled the programme with the 23-valent Pneumococcal Polysaccharide Vaccine (PPV23); for flu, we considered the influenza vaccines recommended by the Joint Committee on Vaccination and Immunisation (JCVI) for the elderly in 2018/19. For COVID-19, we considered the vaccines recommended by the UK Health Security Agency (UKHSA) for the COVID-19 booster vaccination programme in 2021/22. In line with the 2018/19 NHS immunisation schedule [11,12], flu and PD vaccines are modelled in a population of older adults aged 65 and above. As there is currently no approved RSV vaccine in England, we modelled a hypothetical vaccine and assumed the same target population of older adults aged 65+. In 2021/22, the COVID-19 booster vaccine was recommended for adults aged 50 and older [13].

Table 1 summarises effectiveness rates against hospitalisation and coverage rates per age bracket for each vaccination programme. All effectiveness values are averages and are agnostic to specific strains or serotypes. Vaccine effectiveness estimates against hospitalisation following receipt of the influenza vaccine were not available, hence we used effectiveness data against infection [11]. For the RSV vaccine, data on efficacy against hospitalisation in the elderly population aged 65 and above are not yet available. Therefore, we assumed efficacy data against infection [14].

Coverage rates for the flu vaccine are based on annual uptake rates in 2018/19 [11], while coverage rates for the PD vaccines represent total coverage among eligible adults, immunised at any point until 2019 [12]. For the hypothetical RSV vaccine, we assumed it would achieve the same coverage as the PD vaccine. For the COVID-19 booster vaccine we accounted for an increase in coverage over time by applying age-adjusted coverage rates for the period October—December 2021 and for the period January—March 2022 [15].

**Table 1:** Vaccination programme information.

Disease	Age Groups	Vaccine Modelled	Effectiveness	Coverage
Flu	65 years and above	Influenza Vaccine	50% [11] ^1^	72% [11]
PD		Pneumococcal Polysaccharide Vaccine (PPV23)	27% against IPD [16]; 0% against pneumococcal CAP [17]	69% [12] ^2^
RSV		Hypothetical vaccine	50% [14,18] ^1^	69% ^3^
COVID-19	50 years and above	COVID booster vaccine	90% [19]	66.7–91.5% ^4^ [15]

Notes: IPD: invasive pneumococcal disease; CAP: community-acquired pneumonia; ^1^ Effectiveness against infection due to lack of data against infection; ^2^ Total coverage achieved among eligible population; ^3^ Assumption based on PPV23 coverage; ^4^ Variable by age and weeks since vaccination.

Based on routinely collected hospitalisation data from the Hospital Episode Statistics (HES) dataset [20] for relevant years, we extracted the annual number of hospitalisations (measured as finished consultant episodes, FCEs) and the average length of stay (LOS) per hospitalisation due to each disease based on pathogen-specific codes from the international classification of diseases (ICD) (see Tables S2 and S4 in Supplementary Materials). To measure the winter attributable hospitalisations, we weighted the extracted FCE data by an assumed proportion of 67% of the annual hospitalisations occurring from October to March.

We then combined these data with the information on vaccine effectiveness and coverage to estimate the bed-days freed up by vaccination as the prevented hospitalisations by flu, PD, and COVID-19 vaccines, and the preventable hospitalisations by RSV vaccine (See Supplementary Material for detailed methodology). 

We carried out a sensitivity analysis of the vaccine-preventable hospitalisations using plausible lower and upper-bound estimates of key input values (see Table S4 in Supplementary Materials).

### 2.2. Reference Costing versus Opportunity Costing

To value the vaccine-preventable hospitalisations according to the reference costing approach, we valued freed-up bed days by the direct hospitalisation costs of vaccine-preventable hospitalisations based on relevant healthcare resource group (HRG) codes for each ICD code [21,22] (see Tables S3 and S4 in Supplementary Materials). 

To implement the opportunity costing approach, we first converted the bed-days freed-up by vaccination into the number of alternative hospital treatments by dividing them by the average LOS of an alternative hospital treatment [6] (see Table S4 in Supplementary Materials). We then applied two distinct approaches to estimate a likely range of opportunity costs when vaccine-preventable infections require using scarce hospital beds. The range we present is based on Sandmann et al. [5] which considers how opportunity costs differ depending on whether vaccine-preventable hospitalisations are an optimal or suboptimal use of hospital beds compared to alternative hospitalisations. Further explanation of these opportunity costing methodologies and data sources is provided in the Supplementary Material.

- When vaccine-preventable hospitalisations are an optimal use of hospital beds, their opportunity cost is proxied by the forgone benefit from alternative treatment opportunities, which can be quantified in Net Monetary Benefit (NMB) terms. We converted the bed days freed up by vaccination into the number of alternative treatments and valued them in Net Monetary Benefit (NMB) terms. The NMB is estimated using average health gains and average costs from an alternative treatment [6] (see Table S4 in Supplementary Materials).
- When vaccine-preventable hospitalisations are a suboptimal use of hospital beds, their opportunity cost is proxied by the total economic cost, given by the sum of the forgone benefit (NMB) from alternative treatments and the cost of vaccine-preventable hospitalisations.

We carried out a sensitivity analysis of the results obtained through the reference and opportunity costing approaches using plausible lower and upper-bound estimates of key input values (see Table S4 in Supplementary Materials).

### 2.3. What-If Analyses

#### 2.3.1. Impact of Varying the Proportion of Hospitalisations Occurring in the Winter

HES hospitalisation data by ICD code is only available on an annual basis. In the base case analysis, we assumed double hospitalisation rates in the winter (October to March) compared to the rest of the year (67% of annual hospitalisations). However, there is uncertainty regarding the actual proportion of annual hospitalisations due to vaccine-preventable diseases that occur in winter. Further uncertainty is due to the impact of the COVID-19 lockdowns on future infection patterns of seasonal respiratory illnesses (flu, PD, RSV) which have begun to resurge [23,24]. In this what-if analysis, we used a lower bound of 50% of annual hospitalisations and an upper bound of 75%.

#### 2.3.2. Impact of Replacing the Current PPV23 Programme for PD with PCV20 Data

A pneumococcal conjugate 20-valent vaccine (PCV20) is licensed for use in the UK for adults [25]. The current pneumococcal national immunisation programme for adults older than 65 years in England utilises the PPV23 vaccine. The protection offered by PPV23 is considered suboptimal, particularly when compared to a conjugate vaccine that includes a similar number of pneumococcal serotypes [19,26]. Therefore, we modelled the impact of replacing PPV23 with PCV20 to understand the impact of introducing pneumococcal conjugate vaccine to the national immunisation programme.

The PCV20 vaccine was licensed based on randomised-controlled trial immunogenicity data [27], so no published clinical efficacy or effectiveness data exists. PCV20 contains the same components as PCV13. Therefore, we used efficacy and effectiveness data for PCV13 for both invasive pneumococcal disease (IPD) and community acquired pneumonia (CAP), combined with the broader serotype coverage of PCV20. The assumed efficacy values for the PCV20 vaccine are 75% against IPD and 45% against pneumococcal CAP [28].

## 3. Results

### 3.1. Winter Flu, PD and RSV Impact 

#### 3.1.1. Vaccine-Preventable Hospitalisations

**Figure 1:**
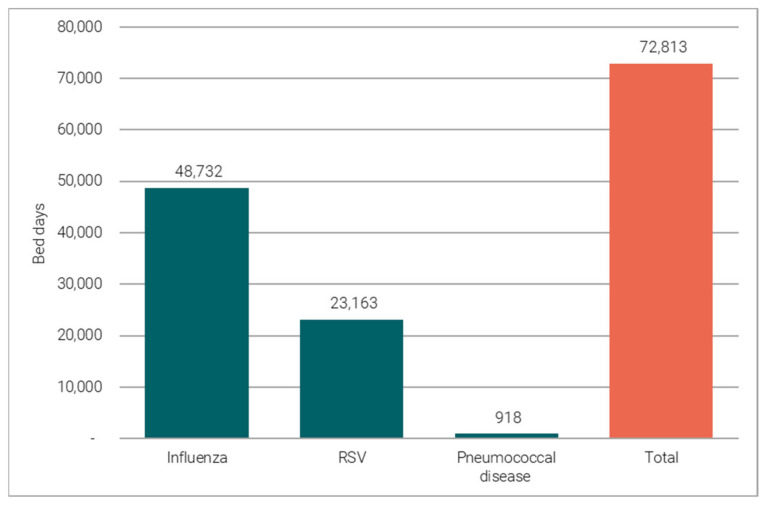
Flu, PD and RSV vaccine-preventable hospitalisations.

In winter, based on pre-COVID-19 disease patterns (2018/19), vaccination programmes for older adults targeting flu, PD and RSV, are estimated to prevent 72,813 (23,992–174,900) bed-days in the winter (October–March) (Figure 1).

#### 3.1.2. Reference Costing versus Opportunity Costing

**Figure 2:**
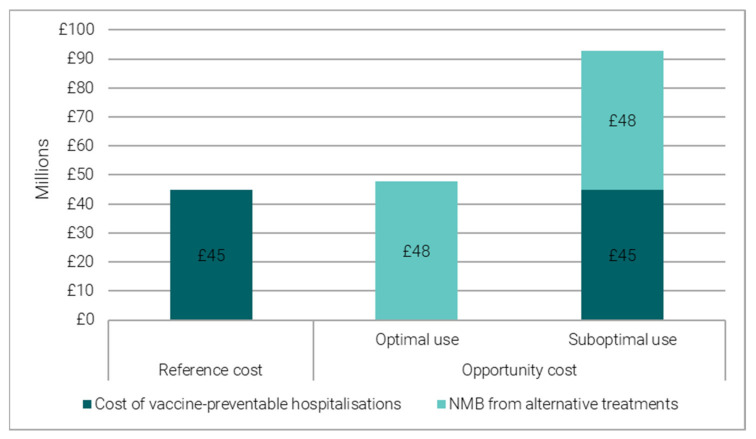
Reference and opportunity cost of flu, PD and RSV vaccine-preventable hospitalisations.

Bed days preventable by flu, PD and RSV vaccination programmes are estimated to save over £45 million (£21–84 million) in direct hospitalisation costs (Figure 2). Specifically, we estimate that about £30 million is saved by flu vaccination programme, £14.5 million by RSV vaccination programme and £0.5 million by PD vaccination programme (Supplementary Materials, Figure S2).

The bed-days freed-up by these vaccination programmes could have been used to admit another 14,533 (3332–53,000) patients to hospital. When allocating beds to vaccine-preventable hospitalisations is optimal compared to alternative bed uses, the benefit generated through the alternative hospitalisations are estimated to have a NMB of £48 million (£3.5–219.5 million) (Figure 2). Specifically, we estimate an NMB of about £32 million from a flu vaccine, £15.4 million from an RSV vaccine and £0.6 million from a PD vaccine (Supplementary Materials, Figure S2).

Due to their urgency, vaccine-preventable hospitalisations may take priority over elective procedures, even though they may generate a lower NMB than other uses of the same bed (i.e., they could be a suboptimal choice). Accounting for this shows that flu, PD, and RSV vaccines would prevent a higher opportunity cost of around £93 million (£24.5–303.5 million) (Figure 2), in the pre-COVID time period of 2018/19.

### 3.2. Winter COVID-19 Impact

#### 3.2.1. Vaccine Preventable Hospitalisations

Based on 2021/22 disease patterns, the COVID-19 booster vaccine is estimated to prevent 2,203,516 (1,346,785–3,323,580) bed days due to COVID-19 infection in the winter (October–March).

#### 3.2.2. Reference Costing versus Opportunity Costing

**Figure 3:**
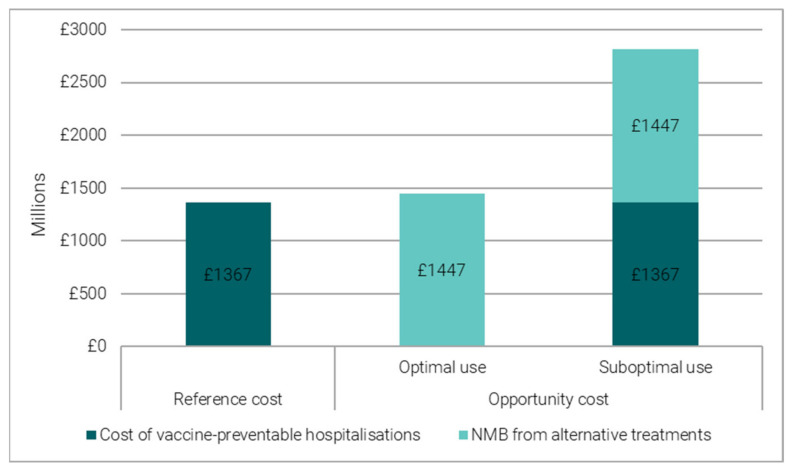
Reference and opportunity cost of COVID-19 vaccine-preventable hospitalisations.

Bed days preventable by the COVID-19 booster vaccination during the winter period 2021/22 were estimated to save over £1.3 billion (£1–2 billion) in direct hospitalisation costs (Figure 3). 

The bed-days freed-up by the COVID-19 booster vaccine could be used to admit another 439,823 (187,053–980,479) patients to hospital. When allocating beds to vaccine-preventable hospitalisations is optimal, the benefits generated through the alternative hospitalisations are estimated to generate a NMB of over £1.4 billion (£200 million–£4 billion) (Figure 3). However, if allocating beds to vaccine-preventable hospitalisations is the sub-optimal choice, the opportunity cost of vaccines is greater. In the case of the COVID booster programme, the opportunity cost would then be around £2.8 billion (£1.2–6 billion) (Figure 3).

### 3.3. What-If Analyses

#### 3.3.1. Impact of Varying the Proportion of Hospitalisations Occurring in the Winter

**Figure 4:**
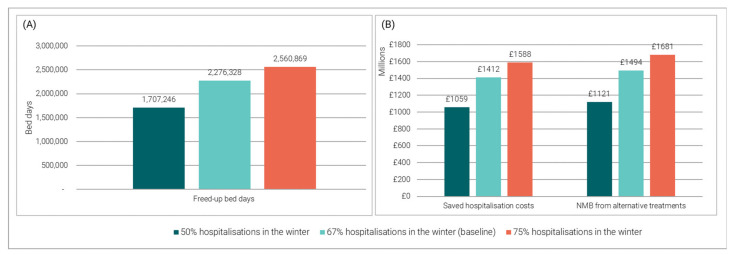
Impact of constant and tripled hospitalisation rates in the winter: (**A**) Vaccine-preventable bed days; (**B**) Saved hospitalization costs and NMB from alternative treatments.

Figure 4 shows the impact of assuming constant and tripled hospitalisation rates compared to the rest of the year on the number of freed-up bed days (panel A), and the saved hospitalisation costs and NMB obtained from alternative treatments (panel B).

Assuming constant hospitalisations across the year (50% of the annual hospitalisations in the winter) would decrease the volume of freed-up bed days, hospitalisation costs averted, and NMB from alternative treatments by approximately 25% compared to the baseline. Assuming a tripling of hospitalisations in the winter (75% of the annual hospitalisations) would increase these estimates by about 13% compared to the baseline.

#### 3.3.2. Impact of Replacing the Current PPV23 Programme for PD with PCV20 Data

Figure 4 shows the impact of replacing the current PPV23 programme for PD with PCV20 on on the number of freed-up bed days (panel A), and the saved hospitalisation costs and NMB obtained from alternative treatments (panel B).

**Figure 5:**
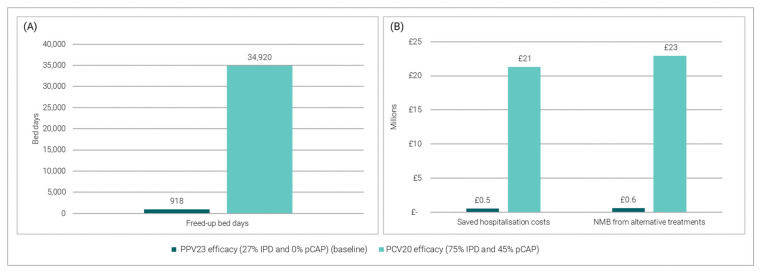
Impact of replacing PPV23 with PCV20: (**A**) Vaccine-preventable bed days; (**B**) Saved hospitalization costs and NMB from alternative treatments.

A PCV20 vaccine could prevent 34,920 bed days. The related hospitalisation savings are estimated at approximately £21 million, and the NMB expected from alternative treatment opportunities is £23 million (Figure 5). Compared to the PPV23 vaccine, assuming the introduction of a PCV20 vaccine increases each modelled outcome by approximately 38 times.

## 4. Discussion

This study estimated the number of hospitalisations that current vaccination programmes against influenza (flu), pneumococcal disease (PD), COVID-19, and a hypothetical Respiratory Syncytial Virus (RSV) vaccination programme, prevent in winter among older adults in England. Based on 2018/19 hospitalisations, the flu, PD and RSV vaccination programmes are likely collectively preventing 72,813 bed days between October and March. Based on 2021/22 hospitalisations, the COVID-19 vaccination programme prevents over 2 million bed days in winter.

It also showed that, under severe excess demand, the value of vaccines is likely to be 1.1–2 times larger (£48–93 million for flu, PD and RSV; and £1.4–2.8 billion for COVID-19) when quantified in terms of their opportunity costs, representing the true cost of scarce beds when they are subject to competing demands, rather than their reference costs.

In a hypothetical scenario where all infections occur at the same time, the four vaccination programmes could collectively prevent over 2.2 million bed days in the winter months (October to March)—with hospitalisation costs saved by avoiding vaccine-preventable hospitalisations amounting to over £1.4 billion, and opportunity costs avoided in the range of £1.5–2.9 billion. Based on our estimates, the COVID-19 booster vaccination programme is a major contributor to this value (approximately 96% of the total). However, the contribution from the other vaccination programmes is not negligible when put into perspective. For example, flu, PD and RSV vaccination among older adults in England are estimated to generate savings of around £45 million from preventing hospitalisations. This value corresponds to approximately 5% of the total UK 2019 healthcare budget allocated to immunisation programmes (£0.9 billion) [29]. As we further highlight below, the most recent winter season (2022/23) saw an increase in hospitalisations for flu compared to the pre-COVID-19 pandemic trends [30], while there is also uncertainty on the future presentation of COVID-19. Therefore, it is likely that our estimates based on pre-COVID-19 disease patterns could underrepresent the value that flu, PD and RSV vaccination programmes will provide in the future.

The opportunity cost estimates are presented as ranges which reflect a diversity of approaches to defining opportunity costs in the economic literature, based on whether vaccine-preventable hospitalisations are optimal or suboptimal compared to alternative uses of hospital beds [5]. Optimality, in this case, is determined by directly comparing the on-average achievable NMB obtained from vaccine-preventable and alternative hospitalisations. Empirically verifying this condition is beyond the scope of this work. However, vaccine-preventable hospitalisations are likely to take priority over other types of elective hospitalisations because, as acute episodes, they are treated urgently. Furthermore, there is evidence suggesting the vaccines included in our analysis could be cost-effective using conventional approaches. As a result, there is considerable support for the assumption that vaccine-preventable hospitalisations may be a suboptimal choice compared to other elective procedures that are not preventable. 

Our results are consistent with Brassel et al. [10] and Sandmann et al. [6] and add to the body of the literature showing that conventional approaches to cost healthcare resources might not appropriately reflect the true opportunity costs prevented by vaccination. Brassel et al. [10] refer to this as the “health system capacity” value of vaccines. They argue that failure to consider this value component may underestimate the total cost-effectiveness of vaccination programmes, especially in settings where the health system is experiencing severe pressure on scarce resources. An incomplete value assessment of vaccines and other preventative treatments may lead to a suboptimal allocation of healthcare prevention budgets.

Findings from this work are timely and relevant. As the pressure experienced by the NHS to address an unprecedented backlog of patients from the COVID-19 pandemic has been compounded by additional demand due to vaccine-preventable seasonal illnesses, allocation decisions regarding prevention budgets in England need to be well-informed. Ensuring sufficient coverage and optimal uptake of highly effective vaccines through national immunisation programmes is critical to alleviating the pressure on the NHS. Based on the evidence provided, decision-makers are recommended to ensure that existing immunisation targets are achieved and programmes are expanded where cost-effective, considering their opportunity costs. 

Results from the hypothetical RSV vaccine are a relevant example of the importance of introducing new vaccination programmes into the national immunisation schedule, as highly efficacious vaccines become available. If recommended by the JCVI, an RSV vaccination programme covering older adults, could prevent an additional 23,163 bed days, save £14 million in hospitalisation costs, and enable other hospitalisations for a value of about £15 million. This study also highlights the importance of timely review by the JCVI of emerging vaccine candidates, which is particularly relevant for the adult PD immunisation programme. PCV20 may provide improved protection compared to the current PD immunisation programme in England using the PPV23 vaccine ([22]. Our what-if analysis shows that by replacing the current vaccine in the PD programme with a PCV20 vaccine, the volume of preventable hospitalisations and related savings may increase substantially (by approximately 38 times).

Overall, our results call for relevant decision makers (e.g., the Department of Health and Social Care, NHS, JCVI, UKHSA) to plan for optimal access to and uptake of available vaccination programmes. Annual flu and PD vaccination programs are well established. However, a more effective higher valent pneumococcal conjugate vaccine could significantly reduce pressure on hospitals. Similarly, maintaining broad eligibility for the COVID autumn booster campaign to high-risk groups is likely to prevent many hospitalisations. Finally, as RSV vaccines are coming to market, rapid review, procurement, programme implementation, and optimal uptake are advised, as tens of thousands of hospitalisations could be prevented.

Our study has limitations. One is that our model is based on retrospective analysis of 2018/19 hospitalisation patterns for flu, PD and RSV and 2021/22 hospitalisations for COVID-19. We assumed these periods to model a winter where seasonal respiratory diseases are circulating again after the COVID-19 lockdowns. While this is a justifiable choice in the absence of more recent data, it has implications for the vaccine-specific contributions to the volume of preventable bed days and their value estimated by our model. As discussed above, the COVID-19 booster vaccine is a major contributor to the model outcomes. Nonetheless, observational data from the most recent 2022/23 winter have shown a surge in hospitalisations and excess deaths due to respiratory diseases like flu and pneumonia compared to previous winters [30]. Therefore, the hospitalisations prevented by non-COVID-19 vaccines may be currently underestimated. Further, the incidence of COVID-19 disease in Europe has showed a decreasing trend since its peak in 2021/22 [31], thanks to effective vaccination campaigns. As a result, in future winters we could expect less asymmetry in the volume of hospitalisations prevented by each vaccination programmes than currently estimated by our model.

The first what-if analysis partially addresses some of the uncertainty, where we varied the proportion of annual hospitalisations occurring in the winter to show the impact of changes in the rate of respiratory infections. This analysis showed that an increase from the baseline of 67% to 75% of annual hospitalisations occurring in the winter would increase the number of vaccine-preventable bed days and their value by 13%. 

Other limitations relate to the methodology used to estimate the NMB of an average hospital treatment. For a discussion of these we refer the reader to Brassel et al. [10].

## 5. Conclusions

This model-based analysis estimates that vaccination programmes for older adults in England against flu, PD and RSV could collectively prevent 72,813 bed days between October and March, and the COVID-19 vaccines could prevent an additional 2 million bed days over this period. Under severe excess demand, their value in opportunity cost terms is likely to be 1.1–2 times larger than their reference costs valuation. 

Understanding and considering opportunity costs is key to ensuring maximum value is obtained from preventative budgets, as the current approaches of reference costing may significantly underestimate the true value of vaccines. In order to build a more resilient system in preparation for the upcoming 2023/24 season, decision-makers should focus on the role of prevention in alleviating pressure on hospitals.

Future research should continue evaluating the volume of vaccine-preventable hospitalisations to understand the impact of potential changes in infection transmission patterns and the average gains from hospital treatments that are used to value the opportunity costs. In addition, methodological work is required to establish how the opportunity costing approach could be considered more formally in healthcare decision-making processes.

## Data Availability

Publicly available datasets were analyzed in this study. This data can be found here: https://digital.nhs.uk/data-and-information/publications/statistical/hospital-admitted-patient-care-activity (accessed on 21 July 2022); https://www.england.nhs.uk/costing-in-the-nhs/national-cost-collection/#archive (accessed on 21 July 2022).

## Supplementary Materials

The following supporting information can be downloaded at: https://www.mdpi.com/article/10.3390/vaccines11050945/s1, Table S1: Modelling Scenario; Table S2: ICD codes by diseases; Table S3: HRG codes by disease; Table S4: Input baseline, lower and upper bound values; Figure S1: Illustrative example of reference costing versus opportunity costing; Figure S2: Vaccine specific contributinos to reference costing and opportunity costing. References [32,33,34,35,36] are cited in the Supplementary Materials.
