# Supplementary material for: Vaccine-Preventable Hospitalisations from Seasonal Respiratory Diseases: What Is Their True Value?
Source: Vaccines (Basel). 2023 May 4;11(5):945. doi: 10.3390/vaccines11050945 (PMC10220863; doi:10.3390/vaccines11050945)
Supplement: Supplementary file 1 [file vaccines-11-00945-s001.zip › vaccines-2344023-supplementary.pdf]

Supplementary Materials

Article: Vaccine-Preventable Hospitalisations from Seasonal Respiratory Diseases: What Is Their True Value?

Authors: Margherita Neri [1,*], Simon Brassel [1], Hannah Schirrmacher [2], Diana Mendes [3], Andrew Vyse [3], Lotte Steuten [1] and Elizabeth Hamson [3]

[1] Office of Health Economics, London SE1 2HB, UK

[2] Triangulate Health, Doncaster DN11 9QU, UK

[3] Pfizer Ltd., Tadworth KT20 7NT, UK

* Correspondence: mneri@ohe.org

1. Vaccine-preventable hospitalisations

To model the number of vaccine-preventable hospitalisations in the winter months (October to March), we considered pre- and during-COVID-19 hospitalisation patterns separately. Specifically, for flu, PD and RSV, we considered pre-COVID-19 hospitalisation patterns from 2018/19. For COVID-19, we considered 2021/22 hospitalisation patterns (Table S1).

TABLE S1: MODELLING SCENARIO

Disease	Scenario	Hospitalisation data
Flu	Estimate the number of hospitalisations pre-COVID that *were prevented* due to vaccination.	Observed hospitalisations pre-COVID (annual HES data 2018/19) [2]
PD	Estimate the number of hospitalisations pre-COVID that *were prevented* due to vaccination.	Observed hospitalisations pre-COVID (annual HES data 2018/19) [2]
COVID-19	Estimate the number of hospitalisations pre-COVID that *were prevented* due to vaccination.	Observed hospitalisations during COVID-19 booster vaccine campaign (annual HES data 2021/22) [2]
RSV	Estimate the number of hospitalisations pre-COVID *that could have been prevented* by vaccination.	Observed hospitalisations pre-COVID (annual HES data 2018/19) [2]

Efficacy and coverage rates for each vaccination programme of our model are summarised in Table S4. All programmes are currently provided by the NHS, except for RSV. Effectiveness data against hospitalisation were not available for all vaccination programmes. For the influenza vaccine we used efficacy data from PHE 2019 reports [3]. The report states that vaccine effectiveness was measured using a test-negative case control design through 5 primary care influenza sentinel swabbing surveillance schemes. Tests are conducted in primary care, therefore the assumed vaccine efficacy rate is against infection. For the RSV vaccine, data on efficacy against hospitalisation in the elderly population aged 65 and above are not yet available. Therefore, we assumed efficacy data against infection [4]. For the PD vaccine (PPV23), we assumed that 3.7% of PD hospitalisations are related to invasive

pneumococcal disease (IPD) and 96.3% are related to pneumococcal community-acquired pneumonia (pCAP) [5,6]. Therefore, we used efficacy values against hospitalisation for both IPD and pCAP [7,8].

To extract hospitalisations and average length of stay (LOS) per hospitalisation due to each disease we first identified the relevant ICD codes. ICD codes for each disease have been identified from the literature and filtered to capture only the codes associated to the relevant pathogen (Table S2). The J18.* code was added to seasonal flu, PD and RSV as it captures a significant bulk of pneumonia admissions with unspecified cause. We used estimates of the number of admissions for respiratory illness observed in England in 2000/01-2007/08 that are explained by influenza and other respiratory pathogens, to determine the share of J18.* admissions that are due to seasonal flu (2%), PD (7.3%) and RSV (5.2%) [9].

TABLE S2: ICD CODES BY DISEASE

Disease	ICD code	Sources
Flu	J10.0, J10.1, J10.8, J11.0, J11.1, J11.8, J18.*	[10]
PD	G00.1, A40.3, J13.X, J18.*	[11]
COVID-19	U07.1, U07.2	[12]
RSV	J12.1, J18.*, J20.5, J21.0	[13]

Using these ICD codes, the number of finished consultant episodes (FCEs) and the mean length of stay (LOS) per FCE are extracted from the HES dataset [2] (Table S4). HES activity data by ICD code is only available on an annual basis. To measure the winter attributable hospitalisations, we weight the extracted FCE data to obtain the proportion of hospitalisations that are attributable to the winter season. We define the winter period as October-March to account for differences in the weeks defining the 'peak season' across pathogens. Infection-specific data or estimates of the proportion of hospitalisations occurring in the winter period are unavailable. As a baseline, we assumed that 67% of the annual hospitalisations occur in the winter and we conducted a what-if analysis assuming different hospitalisation trends across the year (see what-if analysis 1).

For the COVID-19 booster vaccine we accounted for an increase in coverage over time by dividing season-adjusted hospitalisations extracted from annual HES data into two stages. We applied age-adjusted coverage rates to hospitalisations for the period October 2021-December 2021 and for the period January 2022-March 2022, we accounted for an increase in the coverage rate.

Preventable hospitalisations per vaccination programme were calculated based on the scenarios outlined in Table S1Table S. For flu, PD and COVID-19 hospitalisation data includes the impact of vaccination which are currently in place against these infections. Therefore, bed-days freed up by vaccination are estimated as follows:

- Firstly, the number of hospitalisations without the vaccine (counterfactual) are estimated: observed hospitalisations/(1-coverage*efficacy).

- Secondly, the vaccine prevented hospitalisations are calculated: Counterfactual – observed hospitalisations.
- Finally, freed-up bed days per vaccination programme are calculated as: the number of prevented hospitalisations * LOS per preventable outcome.

For RSV, hospitalisation data does not include the impact of RSV as a vaccination programme is currently not in place. Therefore, bed-days freed up by vaccination for RSV are estimated as follows:

- Firstly, the number of hospitalisations if the vaccine had been in place (counterfactual): observed hospitalisations*(1-coverage*efficacy).
- Secondly, the vaccine prevented hospitalisations are calculated: Observed hospitalisations – counterfactual.
- Finally, freed-up bed days per vaccination programme are calculated as: the number of prevented hospitalisations * LOS per preventable outcome.

2. Reference costing versus opportunity costing approach

To extract the required cost data, we used the grouper file to identify the corresponding HRG values for the disease-relevant ICD codes [12]. Using the national cost collection data for 2020/21, [14], the HRG codes were then used to obtain an activity weighted average of the cost per FCE related to the four diseases. The relevant codes for each disease are in Table S3. The activity weighted average of the cost per FCE and HRG codes are in Table S4.

For each disease, the cost of vaccine preventable hospitalisations is estimated as: prevented hospitalisations measured in number of FCEs*Cost per FCE.

TABLE S3 HRG CODES BY DISEASE

Disease	HRG
Flu	DZ11, WH03, DZ23
PD	AA22, WJ06, DZ11, DZ23
COVID-19	DX21, DX11, DX01
RSV	DZ11, DZ23, DZ22

To implement the opportunity costing approach, we first converted the bed-days freed-up by vaccination into the number of alternative hospital treatments by dividing them by the average LOS of an alternative hospital treatment [15]. The resulting number of alternative hospital treatments were valued in Net Monetary Benefit (NMB) terms. To estimate the NMB, the average health gain from an alternative treatment [15] (Table S4) was multiplied by the cost-effectiveness threshold (£20,000 per QALY gained [16]) to quantify the monetised health benefits. The average cost from the alternative treatment [15] (Table S4) was then subtracted to obtain the NMBs.

All input values (Table S4) were derived from Sandmann et al.'s (2018) who estimated the NMB from a non-gastroenteritis hospital admitted patient in England. Of note, our estimates of the NMB from alternative treatments should only exclude the health gains and costs associated with the vaccine-preventable outcome but should include patients with gastroenteritis. However, FCEs relevant to gastroenteritis constitute only 1.13% of the total FCEs in 2021/22 [2,15], hence Sandmann et al.'s [15] estimates are considered the best available proxy for the NMB obtainable from an average alternative hospital patient.

TABLE S4 INPUT BASELINE, LOWER AND UPPER BOUND VALUES

Variable	Value (sensitivity analysis)	Sensitivity analysis and data sources
**Pneumococcal disease (PPV23 vaccine)**		
Vaccine coverage pneumococcal (65 and over)	69.20% (55.36%, 83.04%)	Baseline values varied by ± 20% [17]
Vaccine efficacy pneumococcal IPD (65 and over)	27.00% (17.00%, 35.00%)	[7]
Vaccine efficacy pneumococcal pCAP (65 and over)	0% (0%, 0%)	[8]
Pneumococcal disease hospitalisations attributable to pCAP	96.30% (94.40%-98.20%)	[5,6]
Pneumococcal LOS per FCE, days	4.39 (4.10, 8.19)	Minimum and maximum LOS per FCE of relevant ICD-10 codes [2]
Pneumococcal cost per FCE	£2,678.39 (£2,651.59, £3672.49)	Minimum and maximum HRG value for each disease [14]
**Flu**		
Vaccine coverage flu (65 and over)	72.00% (57.60%, 86.40%)	Baseline values varied by ± 20% [3]
Vaccine efficacy flu (65 and over)	50.00% (39.92%, 59.88%)	Baseline values varied by ± 20% [3]
Flu LOS per FCE, days	3.98 (1.88, 5.55)	Minimum and maximum LOS per FCE of relevant ICD-10 codes [2]
Flu cost per FCE	£2,440.43 (£2,127.31, £2,675.64)	Minimum and maximum HRG value for each disease [14]
**RSV**		
Vaccine coverage RSV (65 and over)	69.20% (55.36%, 83.04%)	Assumed same as PD vaccine
Vaccine efficacy RSV (65 and over)	86.70% (53.80%, 96.50%)	95% confidence interval of the point estimate [4]
RSV LOS per FCE, days	4.26 (3.21, 5.55)	Minimum and maximum LOS per FCE of relevant ICD-10 codes [2]
RSV cost per FCE	£2,648.94 (£1,849.40, £2675.64)	Minimum and maximum HRG value for each disease [14]
**COVID-19**		
Vaccine coverage COVID (50-54) Oct-Dec21	66.70% (66.70%, 74.00%)	

Vaccine coverage COVID (55-59) Oct-Dec21	72.90% (72.90%, 78.70%)	Minimum and maximum coverage per age group [18]
Vaccine coverage COVID (60-64) Oct-Dec21	78.60% (78.60%, 82.60%)	
Vaccine coverage COVID (65-69) Oct-Dec21	84.60% (84.60%, 87.00%)	
Vaccine coverage COVID (70-74) Oct-Dec21	89.30% (89.30%, 90.80%)	
Vaccine coverage COVID (75-79) Oct-Dec21	91.10% (91.10%, 92.50%)	
Vaccine coverage COVID (80-84) Oct-Dec21	89.70% (89.70%, 91.50%)	
Vaccine coverage COVID (85-89) Oct-Dec21	89.70% (89.70%, 91.50%)	
Vaccine coverage COVID (90 and over) Oct-Dec21	89.70% (89.70%, 91.50%)	
Vaccine coverage COVID (50-54) Jan-Mar22	74.00% (66.70%, 74.00%)	
Vaccine coverage COVID (55-59) Jan-Mar22	78.70% (72.90%, 78.70%)	
Vaccine coverage COVID (60-64) Jan-Mar22	82.60% (78.60%, 82,60%)	
Vaccine coverage COVID (65-69) Jan-Mar22	87.00% (84.60%, 87.00%)	
Vaccine coverage COVID (70-74) Jan-Mar22	90.80% (89.30%, 90.80%)	
Vaccine coverage COVID (75-79) Jan-Mar22	92.50% (91.10%, 92.50%)	
Vaccine coverage COVID (80-84) Jan-Mar22	91.50% (89.70%, 91.50%)	
Vaccine coverage COVID (85-89) Jan-Mar22	91.50% (89.70%, 91.50%)	
Vaccine coverage COVID (90 and over) Jan-Mar22	91.50% (89.70%, 91.50%)	
Vaccine efficacy COVID (50 and over) up to 10 weeks	90.00% (85.00%, 95.00%)	[19,20]
COVID-19 LOS per FCE, days	4.47 (3.72, 4.48)	Minimum and maximum LOS per FCE of relevant ICD-10 codes [2]
COVID-19 cost per FCE	£3,655.51 (£2,771.06, £4,506.88)	Minimum and maximum HRG value for each disease [14]
**Other input value estimates**		
Backlog cost	£1,491.00 (£1,766.00, £1,058.00)	Lower and upper quartiles of the point estimate
Backlog LOS	5.01 (7.20, 3.30)	[15]
QALYs Non-gastroenteritis cases with chronic conditions	0.239 (0.142, 0.260)	95% confidence interval of the point estimate [15]

3. Valuing vaccine preventable hospitalisations

Due to the scarcity of hospital beds, treating a patient with a vaccine-preventable infection (e.g. flu) means losing the opportunity to treat other patients requiring care. These circumstances reflect the standard economic decision problem of value maximisation under

constrained resources, where each decision carries an opportunity cost. There are multiple ways of calculating opportunity costs. The available approaches range between considering the cost incurred for the chosen treatment and different measures of what is lost by forgoing alternative treatment opportunities [1].

The range of opportunity costs presented in this paper is based on the proposal by Sandmann et al. [1] to consider how opportunity costs differ depending on whether vaccine-preventable hospitalisations represent an optimal or suboptimal use of a bed. This range reflects a diversity of opportunity cost definitions, ranging from the narrower consideration of *forgone benefits from alternative treatment opportunities* to a broader consideration of *full economic costs*.

An example is given in Figure S1, where we consider the opportunity cost of allocating a hospital bed to either patient A or patient B. In this example, patient A represents the optimal treatment choice, as treating them would generate more benefits relative to related costs than in the case of treating patient B.

According to the proposal by Sandmann et al. [1], if we choose to treat Patient A, the opportunity costs are the forgone net benefits from patient B. Net monetary benefits (NMBs) are a relevant metric of net health for decision-makers intending to maximise health as they represent the monetary value of a treatment's health gains minus the treatment costs. However, if we choose to treat patient B, the opportunity costs equal the full economic costs, including the forgone NMB from patient A plus the cost incurred to treat patient B. Economic costs are a more comprehensive measure of what is lost.

Today, the most common approach to value healthcare resources considers the direct costs of the treatment for which they are used (e.g. the cost of treating patient A when hospital bed is allocated to them). This so-called reference (or accounting) costing approach only proxies the opportunity costs correctly under very restrictive and unrealistic conditions, such as perfectly competitive markets [1].

FIGURE S1 ILLUSTRATIVE EXAMPLE OF REFERENCE COSTING VERSUS OPPORTUNITY COSTING

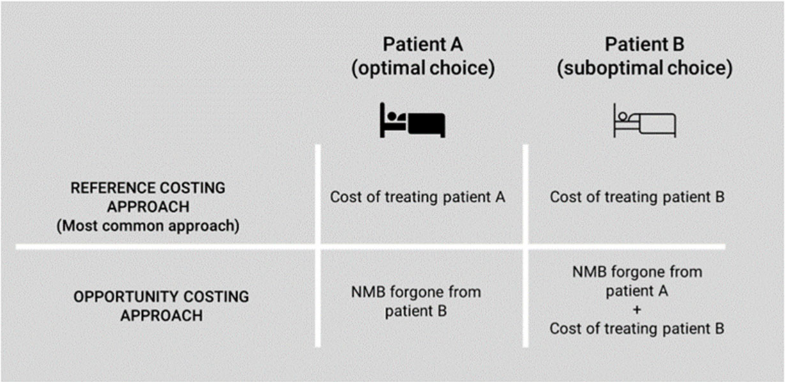

4. Additional figures

FIGURE S2 VACCINE-SPECIFIC CONTRIBUTIONS TO REFERENCE COSTING AND OPPORTUNITY COSTING

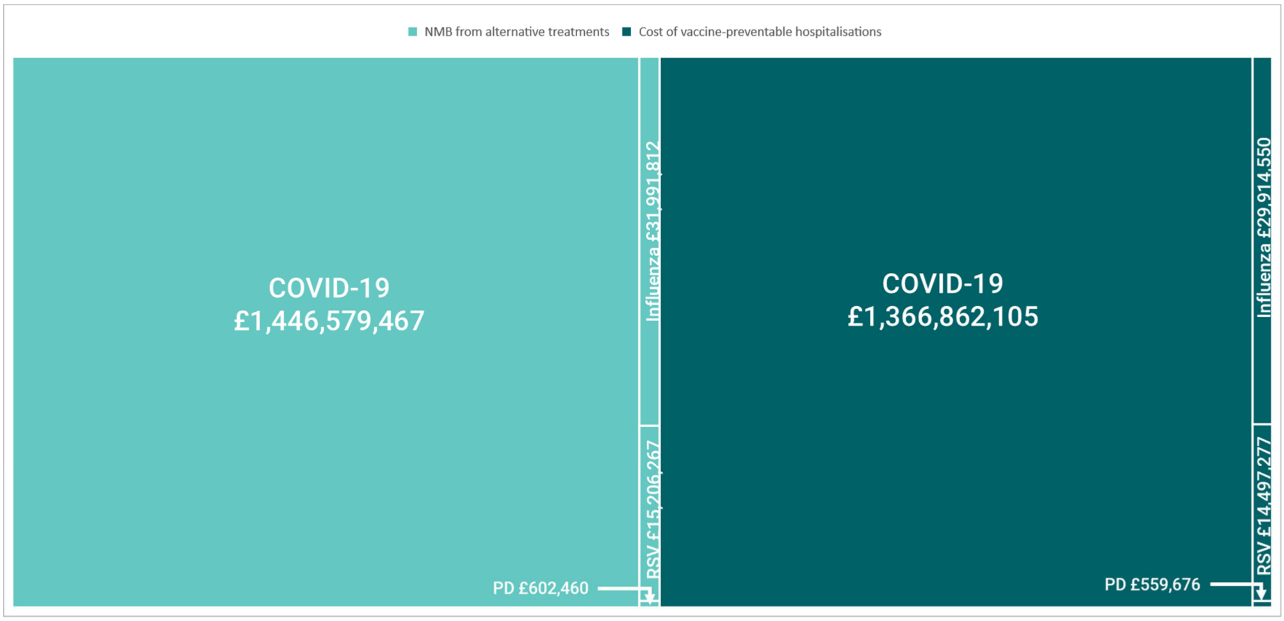

5. References

1. Sandmann, F.G.; Robotham, J.V.; Deeny, S.R.; Edmunds, W.J.; Jit, M. Estimating the opportunity costs of bed-days. Health Econ. 2018, 27, 592–605. https://doi.org/10.1002/hec.3613.
2. NHS Digital. Hospital Admitted Patient Care Activity. 2022. Available online: https://digital.nhs.uk/data-and-information/publications/statistical/hospital-admitted-patient-care-activity (Accessed on 11 March 2022).
3. PHE. Surveillance of influenza and other respiratory viruses in the UK Winter 2018 to 2019. Public Health England, London, United Kingdom, 2019. Available online: https://assets.publishing.service.gov.uk/government/uploads/system/uploads/attachment_data/file/839350/Surveillance_of_influenza_and_other_respiratory_viruses_in_the_UK_2018_to_2019-FINAL.pdf (Accessed on: 6 July 2021).
4. Zeevat, F.; Luttjeboer, J.; Paulissen, J.; van der Schans, J.; Beutels, P.; Boersma, C.; Postma, M. Exploratory analysis of the economically justifiable price of a hypothetical RSV vaccine for older adults in the Netherlands and the United Kingdom. *J. Infect. Dis.* **2022**, *226*, S102–S109.
5. Pick, H.; Daniel, P; Rodrigo, C; Bewick, T; Ashton, D; Lawrence, H; et al. Pneumococcal serotype trends, surveillance and risk factors in UK adult pneumonia, 2013-18. *Thorax*. **2020** Jan;75(1):38–49.
6. Mugwagwa, T.; Averin, A.; Atwood, M.; Sato, R.; Vyse, A.; Campling, J.; Weycker, D.; Slack, M.; Ellsbury, G.; Mendes, D. Public health and budgetary impact of 20-valent pneumococcal conjugate vaccine for adults in England. *Expert Rev. Vaccines* **2022**, *21*, 1331–1341. https://doi.org/10.1080/14760584.2022.2104250.
7. Djennad, A.; Ramsay, M.E.; Pebody, R.; Fry, N.K.; Sheppard, C.; Ladhani, S.N.; Andrews, N.J. Effectiveness of 23-Valent Polysaccharide Pneumococcal Vaccine and Changes in Invasive Pneumococcal Disease Incidence from 2000 to 2017 in Those Aged 65 and over in England and Wales. *eClinicalMedicine* **2018**, *6*, 42–50. https://doi.org/10.1016/j.eclinm.2018.12.007.
8. Lawrence, H.; Pick, H.; Baskaran, V.; Daniel, P.; Rodrigo, C.; Ashton, D.; Edwards-Pritchard, R.C.; Sheppard, C.; Eletu, S.D.; Litt, D.; et al. Effectiveness of the 23-valent pneumococcal polysaccharide vaccine against vaccine serotype pneumococcal pneumonia in adults: A case-control test-negative design study. *PLoS Med*. **2020**, *17*, e1003326. https://doi.org/10.1371/journal.pmed.1003326.
9. Cromer, D; Hoek, .A.J van; Newall, .A.T.; Pollard, A.J.; Jit, M. Burden of paediatric respiratory syncytial virus disease and potential effect of different immunisation strategies: a modelling and cost-effectiveness analysis for England. The Lancet Public Health, United Kingdom, 2017 Aug 1;2(8):e367–74.
10. Franklin, B.; Hochlaf, D. AN ECONOMIC ANALYSIS OF FLU VACCINATION [Internet]. The International Longevity Centre: London, United Kingdom, 2018 Available online: https://ilcuk.org.uk/wp-content/uploads/2018/07/An_economic_analysis_of_flu_vaccination_-_ILC-UK.pdf (Accessed on: 7 October 2022).
11. Delgleize, E.; Leeuwenkamp, O.; Theodorou, E.; Van de Velde, N. Cost-effectiveness analysis of routine pneumococcal vaccination in the UK: a comparison of the PHiD-CV vaccine and the PCV-13 vaccine using a Markov model. BMJ Open. 2016 Nov;6(11):e010776.
12. NHS Digital. HRG4+ 2020/21 Local Payment Grouper V2 (COVID-19). 2021. Availale online: https://digital.nhs.uk/services/national-casemix-office/downloads-groupers-and-tools/hrg4-2020-21-local-payment-grouper-v2-covid-19 (accessed 7 October 2022).
13. Sharp, A.; Minaji, M.; Panagiotopoulos, N.; Reeves, R.; Charlett, A.; Pebody, R. Estimating the burden of adult hospital admissions due to RSV and other respiratory pathogens in England. *Influenza Other Respir. Viruses* **2022**, *16*, 125–131. https://doi.org/10.1111/irv.12910.

14. NHS England. National Schedule of NHS Costs. 2021. Available online: https://view.officeapps.live.com/op/view.aspx?src=https%3A%2F%2Fwww.england.nhs.uk%2Fwp-content%2Fuploads%2F2022%2F07%2F2_National_schedule_of_NHS_costs_FY20-21.xlsx&wdOrigin=BROWSELINK (accessed 6 December 2022).
15. Sandmann, F.G.; Shallcross, L.; Adams, N.; Allen, D.J.; Coen, P.G.; Jeanes, A.; Kozlakidis, Z.; Larkin, L.; Wurie, F.; Robotham, J.V.; et al. Estimating the Hospital Burden of Norovirus-Associated Gastroenteritis in England and Its Opportunity Costs for Nonadmitted Patients. *Clin. Infect. Dis.* **2018**, *67*, 693–700. https://doi.org/10.1093/cid/ciy167.
16. NICE. Assessing cost-effectiveness | The guidelines manual. National Institute for Health and Care Excellence. National Institute for Health and Care Excellence, 2012 Available from: https://www.nice.org.uk/process/pmg6/chapter/assessing-cost-effectiveness. (Accessed on: 6 December 2022).
17. PHE. Pneumococcal Polysaccharide Vaccine (PPV) coverage report, England, April 2018 to March 2019. Public Health England, London, United Kingdom, 2019. Available online: https://webarchive.nationalarchives.gov.uk/ukgwa/20211106003307/https:/www.gov.uk/government/publications/pneumococcal-polysaccharide-vaccine-ppv-vaccine-coverage-estimates (Accessed on: 7 October 2022).
18. UKHSA. *Weekly national Influenza and COVID-19 surveillance report Week 14 report (up to week 13 data) 7 April 2022*; UK Health Security Agency, London, United Kingdom, 2022. Available online: https://assets.publishing.service.gov.uk/government/uploads/system/uploads/attachment_data/file/1067092/Weekly_Flu_and_COVID-19_report_w14.pdf
19. Mendes, D.; Chapman, R.; Aruffo, E.; Gal, P.; Nguyen, J.L.; Hamson, L.; Di Fusco, M.; Czudek, C.; Yang, J. Public health impact of UK COVID-19 booster vaccination programs during Omicron predominance. *Expert Rev. Vaccines* **2023**, *22*, 90–103
20. UKHSA. *COVID-19 vaccine surveillance report Week 12; 24 March 2022*. UK Health Security Agency, London, United Kingdom, 2022. Available Online: https://assets.publishing.service.gov.uk/government/uploads/system/uploads/attachment_data/file/1063023/Vaccine-surveillance-report-week-12.pdf
